# Supplementary material for: Exploratory Full-Field Mechanical Analysis across the Osteochondral Tissue—Biomaterial Interface in an Ovine Model
Source: Materials (Basel). 2020 Sep 4;13(18):3911. doi: 10.3390/ma13183911 (PMC7559087; doi:10.3390/ma13183911)
Supplement: Supplementary file 1 [file materials-13-03911-s001.pdf]

materials

MDPI

Supplementary

Exploratory Full-Field Mechanical Analysis across the Osteochondral Tissue—Biomaterial Interface in an Ovine Model

Jeffrey N. Clark [1,2], Agathe Heyraud [2], Saman Tavana [1], Talal Al-Jabri [3], Francesca Tallia [2], Brett Clark [4], Gordon W. Blunn [5], Justin P. Cobb [3], Ulrich Hansen [1], Julian R. Jones [2] and Jonathan R.T. Jeffers [1,*]

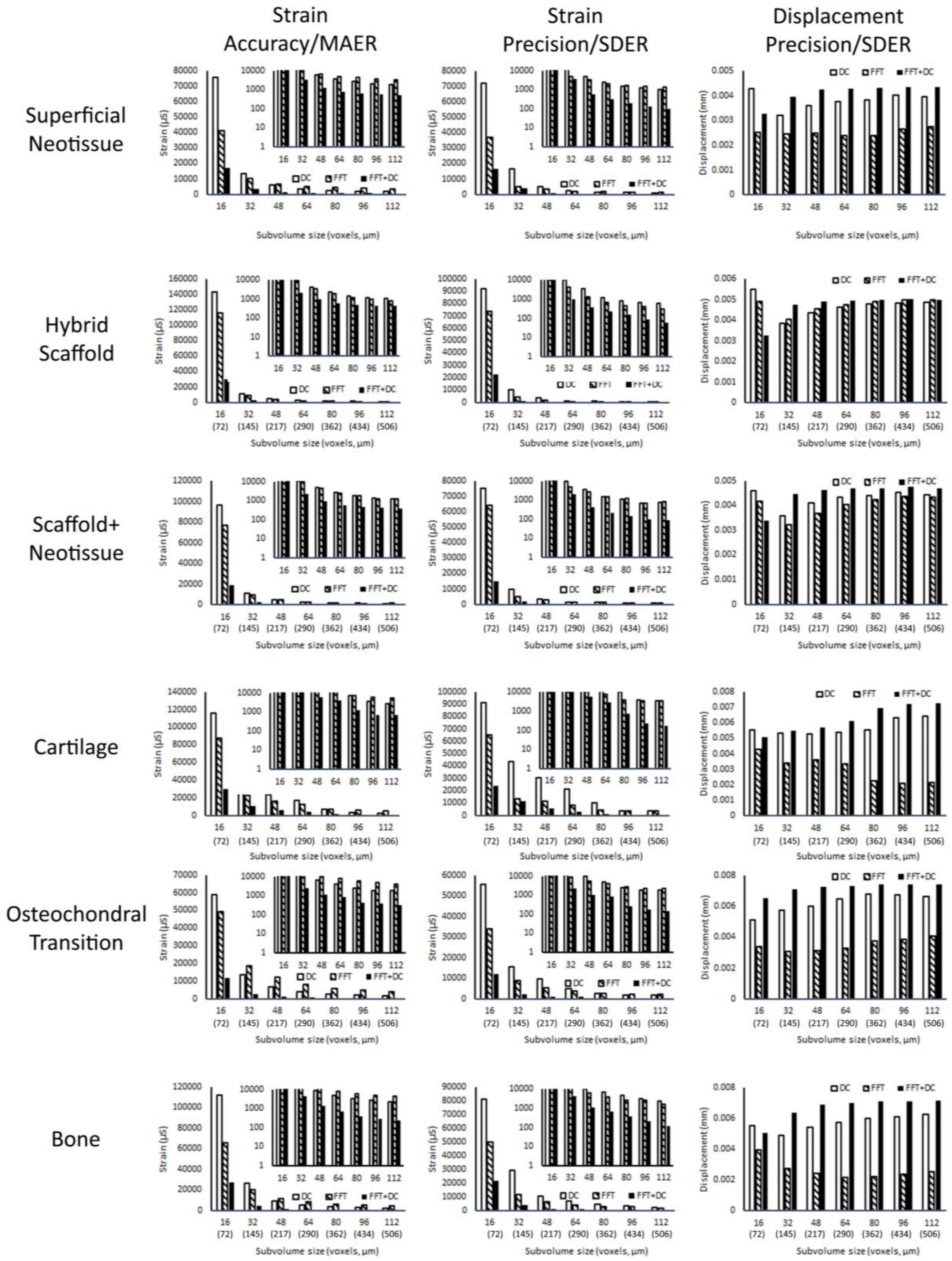

Figure 1. Strain and displacement errors, across a range of subvolume sizes, for each of the tissue components on the sample (n = 1), derived under constant strain conditions for in situ mechanical tested during micro-CT scanning. Please note the logarithmic scale for the inset panels.

© 2020 by the authors. Submitted for possible open access publication under the terms and conditions of the Creative Commons Attribution (CC BY) license (http://creativecommons.org/licenses/by/4.0/).
